# Ambulatory Activity and Risk of Premature Mortality Among Young and Middle-aged American Indian Individuals

**DOI:** 10.1001/jamanetworkopen.2023.11476

**Published:** 2023-05-04

**Authors:** Amanda M. Fretts, David S. Siscovick, Kimberly Malloy, Colleen M. Sitlani, Ana Navas-Acien, Ying Zhang, Jason Umans, Shelley Cole, Lyle G. Best, Barbara V. Howard

**Affiliations:** 1 Department of Epidemiology, University of Washington, Seattle; 2 New York Academy of Medicine, New York; 3 Department of Biostatistics and Epidemiology, University of Oklahoma Health Sciences Center, Oklahoma City; 4 Department of Medicine, University of Washington, Seattle; 5 Department of Environmental Health Sciences, Columbia University, New York, New York; 6 MedStar Health Research Institute, Washington, DC; 7 Texas Biomedical Research Institute, San Antonio, Texas; 8 Missouri Breaks Industries Research Inc, Eagle Butte, South Dakota; 9 Georgetown–Howard Universities Center for Translational Sciences, Washington, DC

## Abstract

**Importance:**

To our knowledge, no published studies have investigated the association of ambulatory activity with risk of death among young and middle-aged American Indian individuals. The burden of chronic disease and risk of premature death is higher among American Indian individuals than among the general US population, so better understanding of the association of ambulatory activity with risk of death is needed to inform public health messaging in tribal communities.

**Objective:**

To examine the association of objectively measured ambulatory activity (ie, steps per day) with risk of death among young and middle-aged American Indian individuals.

**Design, Setting, and Participants:**

The ongoing longitudinal Strong Heart Family Study (SHFS) is being conducted with participants aged 14 to 65 years in 12 rural American Indian communities in Arizona, North Dakota, South Dakota, and Oklahoma and includes up to 20 years of follow-up (February 26, 2001, to December 31, 2020). This cohort study included SHFS participants who had available pedometer data at baseline. Data analysis was performed on June 9, 2022.

**Exposures:**

Objectively measured ambulatory activity at baseline.

**Main Outcomes and Measures:**

Outcomes of interest were total and cardiovascular-related mortality. Mixed-effects Cox proportional hazards regression was used to estimate hazard ratios for risk of death, with entry at the time of the pedometer assessment and time at risk until death or the latest adjudicated date of follow-up.

**Results:**

A total of 2204 participants were included in this study. Their mean (SD) age was 41.0 (16.8) years; 1321 (59.9%) were female and 883 (40.1%) were male. During a mean follow-up of 17.0 years (range, 0-19.9 years), 449 deaths occurred. Compared with participants in the lowest quartile of steps per day (<3126 steps), individuals in the upper 3 quartiles of steps per day had lower risk of mortality, with hazard ratios of0.72 (95% CI, 0.54-0.95) for the first quartile, 0.66 (95% CI, 0.47-0.93) for the second quartile, and 0.65 (95% CI, 0.44-0.95) for the third quartile after adjustment for age, sex, study site, education, smoking status, alcohol use, diet quality, body mass index, systolic blood pressure, prevalent diabetes, prevalent cardiovascular disease, biomarker levels (fibrinogen, low-density lipoprotein cholesterol, and triglycerides), medication use (hypertensive or lipid-lowering agents), and self-reported health status. The magnitude of the hazard ratios was similar for cardiovascular mortality.

**Conclusions and Relevance:**

In this cohort study, American Indian individuals who took at least 3126 steps/d had a lower risk of death compared with participants who accumulated fewer steps per day. These findings suggest that step counters are an inexpensive tool that offers an opportunity to encourage activity and improve long-term health outcomes.

## Key Points

**Question:**

Is objectively measured ambulatory activity (ie, steps per day) associated with risk of death among young and middle-aged American Indian individuals who reside in rural communities?

**Findings:**

In this cohort study of 2204 American Indian individuals aged 14 to 65 years, there were 449 deaths during a mean 17-year follow-up. Participants who accumulated at least 3126 steps/d had a 28% to 35% lower risk of death compared with those who accumulated fewer steps per day.

**Meaning:**

These findings suggest that physical inactivity may be associated with mortality among young and middle-aged American Indian individuals and that physical activity outreach programs that target inactive individuals are needed.

## Teaser

This cohort study examines the association of objectively measured ambulatory activity with risk of premature death among young and middle-aged American Indian individuals.

## Introduction

Physical activity is a core component of a healthy lifestyle. Recommended physical activity levels are based on findings from a large body of epidemiologic studies and clinical trials that consistently demonstrate the association of physical activity with decreased risk of many chronic diseases.^1,2^ Findings from these studies have informed US guidelines, such as the 2018 Physical Activity Guidelines for Americans, which recommend that individuals perform at least 150 minutes of moderate-intensity physical activity per week to achieve optimal health.^3,4^

The intensity and duration of physical activity does not directly translate into daily “steps” needed to achieve health benefits. A goal of 10 000 steps/d has been used in many studies targeting a reduction in cardiovascular risk factors,^5,6,7,8,9^ but research supporting this step guideline is limited.^10,11^ A 2018 report from the Physical Activity Guidelines Advisory Committee^12^ indicates that health benefits may be achieved with the accumulation of as few as 7000 steps/d.

Most studies that have assessed associations of ambulatory activity (ie, steps per day) with risk of death have focused on older adults who reside in urban or suburban communities.^13,14,15,16,17,18^ It is unclear if these findings are generalizable to younger populations, as the threshold of activity needed to achieve health benefits in older individuals may be different than that in middle-aged or younger populations. Additionally, individuals who reside in urban or suburban areas may have different activity patterns than those who reside in rural communities. In support of the 2018 Physical Activity Guidelines for Americans, a recently published study reported that young and middle-aged, urban-dwelling Black and White participants who accumulated 7000 steps/d or more had a 70% lower risk of death than participants who accumulated fewer steps.^19^ To our knowledge, no published studies have elucidated the association of ambulatory activity with risk of death in rural populations in general or among young and middle-aged American Indian individuals in particular. The burden of chronic disease and risk of premature death among American Indian individuals is higher than in the general US population, so better understanding of the association of ambulatory activity with risk of death is needed to inform public health messaging. Because walking is the most common form of physical activity in many American Indian communities,^20^ characterization of risk using steps per day allows for public health messaging that is easy to understand. The overall goal of the present study was to examine the association of objectively measured ambulatory activity with risk of death among American Indian individuals from rural communities.

## Methods

### Study Design and Population

The Strong Heart Study was started in 1988 to better understand risk factors for cardiovascular disease (CVD) among middle-aged or older American Indian individuals. The study was expanded in 2001 to include family members of original participants to allow analyses of the heritability of cardiovascular risk factors and diseases. The Strong Heart Family Study (SHFS) is an ongoing population-based, longitudinal cohort study of risk factors for CVD conducted in 12 American Indian communities in Arizona, North Dakota, South Dakota, and Oklahoma. The SHFS included 2 examinations: one at baseline (February 26, 2001, to September 30, 2003) and the other at follow-up (May 5, 2006, to December 8, 2009). Details of the study have been described previously.^21^ Briefly, 1122 male individuals and 1658 female individuals from 92 large families completed a baseline examination. In 2006 to 2009, 91% of participants who completed the baseline examination participated in a follow-up examination. In addition to information collected at the study examinations, incidences of cardiovascular events and death were assessed in the SHFS by ongoing surveillance via annual phone interviews and medical record reviews through December 31, 2020. The institutional review board from each Indian Health Service region and all communities approved the study, and written informed consent was obtained from participants. The study followed the Strengthening the Reporting of Observational Studies in Epidemiology (STROBE) reporting guideline.

For the current cohort study, we included SHFS participants aged 14 to 65 years who completed the pedometer assessment at baseline. Because it has been shown that 3 days of pedometer data are sufficient to confidently estimate usual physical activity levels,^22,23^ participants who had fewer than 3 days of pedometer data were excluded.

### Data Collection

Each SHFS examination included a standardized personal interview, physical examination, and laboratory workup.^21,24^ Information regarding previous and current medical conditions, education, smoking status, and past-year diet was collected. Anthropometric measurements were obtained, in which the participant wore lightweight clothing and no shoes. Body mass index (BMI) was calculated as body weight in kilograms divided by height in meters squared. Waist circumference was measured at the umbilicus while the participant was in a supine position. Body fat percentage was estimated using bioelectrical impedance. Blood pressure was measured 3 times on the right arm using standard mercury sphygmomanometers after 5 minutes of rest; the means of the second and third systolic and diastolic measurements were used in this analysis. Blood samples were collected after a 12-hour overnight fast and were stored at −80 °C. Plasma glucose, triglyceride, low-density lipoprotein (LDL) cholesterol, and high-density lipoprotein (HDL) cholesterol levels were measured using enzymatic methods.^21,24^

### Ambulatory Activity Assessment (Steps per Day)

The AE120 pedometer (Accusplit) was used to measure daily ambulatory activity at baseline. This pedometer has been shown to be both reliable and valid.^25,26,27,28,29,30,31,32^

The SHFS participants were instructed to wear the pedometer on their hip during waking hours for 7 consecutive days, except while bathing or swimming. Participants were issued a physical activity diary and instructed to record the following: (1) the time the pedometer was put on each morning; (2) the number of steps taken per day; (3) the time the pedometer was removed each evening; (4) whether the pedometer was taken off at any time during the day and, if so, why; and (5) the length of time that the pedometer was off (in minutes or hours). At the end of the 7-day period, participants returned the diary to the SHFS investigators.

### Mortality Assessment

Deaths were adjudicated by the Strong Heart Study Morbidity and Mortality Committee based on information from medical records, death certificates from state health departments, review of the National Death Index, autopsy and coroner reports, local review of obituaries, and/or interviews with next of kin through December 31, 2020. The primary outcome of interest was all-cause (total) mortality. Secondarily, we assessed the association of steps per day with mortality from CVD (ie, deaths from myocardial infarction, coronary heart disease, heart failure, or stroke).

### Statistical Analysis

The primary physical activity measure used for analyses was the mean number of steps taken per day during the time that the pedometer was worn. Steps per day were examined both categorically (in quartiles) and continuously (using cubic splines).

Mixed-effects Cox proportional hazards regression was used to estimate the hazard ratio of death, with entry at the time of pedometer assessment and time at risk until death or the latest adjudicated date of follow-up. To account for the family-based sampling of the SHFS, we utilized random-effects models that clustered on family. Covariates were selected a priori. Three models were fit. The first was a crude model that adjusted for baseline age (in years), sex (male or female), and study site (Oklahoma, Arizona, North Dakota, or South Dakota). The second model additionally adjusted for other baseline sociodemographic factors and health behaviors, including education (in years), smoking status (never, former, or current), and alcohol use and diet quality (both as assessed with the Alternative Healthy Eating Index; linear). The third model additionally adjusted for other baseline factors that may either confound or mediate (eg, in the causal pathway) associations of ambulatory activity with mortality, including BMI (linear), systolic blood pressure (linear), prevalent diabetes (yes or no), prevalent CVD (yes or no), biomarker levels (fibrinogen, LDL cholesterol, and triglycerides; linear), medication use (hypertensive or lipid-lowering agents; yes or no), and self-reported health status (excellent, very good, or good vs fair or poor).

Multiple imputations were used (20 replicates) to address occasional missing values for covariates (<3% missingness for all covariates) using information on age, sex, study site, education, smoking status, alcohol use, diet quality, BMI, systolic blood pressure, LDL cholesterol level, prevalent CVD, and prevalent diabetes. In exploratory analyses, we examined whether associations of steps per day with mortality differed by sex, age, study site, BMI, or diabetes status. Likelihood ratio tests were used to evaluate the statistical significance of the multiplicative interaction term for each factor with steps per day modeled using covariates for the fully adjusted model (model 3). Because it is possible that participants may have had days with unusually high or low steps per day during the time period that the pedometer was worn, we examined the effect of excluding 2 days of activity—the days with the minimum and maximum accumulated steps—in estimating the mean number of steps taken per day in sensitivity analyses. To address potential reverse causation, we repeated all analyses excluding participants who died during the first 2 years of follow-up. We also ran sensitivity analyses that included only participants with self-reported health as excellent, very good, or good at baseline and without CVD to address potential confounding by underlying health status. The significance level of the hypothesis test was set as 2-tailed *P* = .05.

Statistical analyses were conducted using Stata, version 16.0 (StataCorp LLC). Data analysis was performed on June 9, 2022.

## Results

The analytic sample included 2204 SHFS participants with available pedometer data. Their mean (SD) age was 41.0 (16.8) years, 1321 (59.9%) were female and 883 (40.1%) were male, and their mean (SD) BMI was 31.2 (7.4). A total of 13 participants (0.6%) wore the pedometer for 3 days, 125 (5.7%) for 4 days, 58 (2.6%) for 5 days, 125 (5.7%) for 6 days, and 1883 (85.4%) for 7 days. In general, the mean number of steps per day decreased with age, and male participants had higher step counts than female participants. Among participants aged 40 years or younger, male individuals had a median of 7176 steps/d (IQR, 5004-9996) and female individuals had a median of 5196 steps/d (IQR, 3491-7445). Among participants aged older than 40 years, male individuals had a median of 5354 steps/d (IQR, 3523-7993) and female individuals had a median of 3496 steps/d (IQR, 2254-5531). In total, 1539 participants (69.8%) accumulated fewer than 7000 steps/d, 396 participants (18.0%) accumulated 7000-9999 steps/d, and 269 (12.2%) accumulated 10 000 steps/d or more. For comparison with thresholds used in other cohort studies, 365 SHFS participants (16.6%) accumulated more than 9100 steps/d (median, 5085 steps/d; mean, 5841 steps/d). The range of steps per day was 142 to 3125 for the first quartile, 3126 to 5085 for the second quartile, 5086 to 7572 for the third quartile, and 7573 to 31 893 for the fourth quartile.

Baseline characteristics of the study participants according to category of steps are shown in Table 1. Participants who accumulated more steps per day were younger, were more likely to be male, and had a lower BMI, lower body fat percentage, and smaller waist circumference than participants who accumulated fewer steps. Additionally, participants who took more steps per day were less likely to have prevalent diabetes or CVD, were less likely to use hypertensive medication, had lower systolic blood pressure and lower plasma fibrinogen and triglyceride levels, and had higher self-reported health status than participants who took fewer steps. There were no differences in participant education, current smoking status, HDL cholesterol level, or diet quality according to steps per day.

**Table 1.** Baseline Characteristics of Strong Heart Family Study Participants Overall and According to Ambulatory Activity Quartile^a^

Characteristic	Total (N = 2204)	Ambulatory activity quartile, steps/d			
		<3126 (n = 551)	3126-5085 (n = 551)	5086-7572 (n = 551)	≥7573 (n = 551)
Age, y, mean (SD)	41.0 (16.8)	50.0 (17.7)	42.3 (16.0)	37.6 (14.8)	34.2 (14.1)
Sex					
Female	1321 (59.9)	424 (77.0)	366 (66.4)	308 (55.9)	223 (40.5)
Male	883 (40.1)	127 (23.0)	185 (33.6)	243 (44.1)	328 (59.5)
Education, mean (SD), y	12.3 (2.3)	12.1(2.4)	12.6 (2.2)	12.4 (2.3)	12.3 (2.2)
Smoking status					
Never	866 (39.3)	207 (37.6)	222 (40.3)	195 (35.4)	241 (43.8)
Former	538 (24.4)	167 (30.3)	125 (22.7)	131 (23.8)	114 (20.7)
Current	802 (36.4)	177 (32.1)	204 (37.0)	225 (40.8)	196 (35.5)
BMI, mean (SD)	31.2 (7.4)	34.4 (8.1)	31.0 (7.2)	30.5 (6.8)	29.0 (6.3)
Waist circumference, mean (SD), in	40.2 (7.0)	43.5 (7.4)	40.0 (6.6)	39.3 (6.4)	37.9 (6.5)
Body fat, mean (SD), %	36.0 (10.0)	41.2 (9.1)	36.8 (9.4)	35.1 (9.6)	31.1 (9.3)
Disease prevalence					
Diabetes	414 (18.8)	187 (33.9)	107 (19.5)	79 (14.3)	39 (7.1)
CVD	110 (5.0)	55 (10.0)	28 (5.1)	12 (2.2)	15 (2.7)
Systolic blood pressure, mean (SD), mm Hg	123.1 (16.6)	127.4 (18.0)	123.9 (16.5)	120.9 (15.2)	120.0 (15.5)
Biomarker level, mean (SD), mg/dL					
HDL cholesterol	51.8 (14.5)	51.1 (14.8)	52.3 (15.4)	51.5 (14.4)	52.5 (13.6)
LDL cholesterol	100.1 (30.3)	97.4 (29.2)	100.4 (29.9)	101.6 (29.9)	101.1 (32.1)
Triglycerides	165.9 (182.3)	190.3 (248.5)	175.2 (154.0)	161.1 (196.1)	137.2 (87.5)
Fibrinogen	378.2 (87.1)	415.4 (97.0)	384.6 (84.2)	361.7 (75.9)	350.9 (75.6)
Medication use					
Lipid-lowering agent	185 (8.4)	66 (12.0)	51 (9.3)	27 (4.9)	31 (5.6)
Hypotensive agent	461 (20.9)	172 (31.2)	118 (21.5)	83 (15.1)	60 (10.9)
Self-rated fair or poor health	480 (21.8)	200 (36.3)	122 (22.1)	84 (15.2)	74 (13.4)
Ambulatory activity, mean (SD), steps/d	5841.1 (3901.7)	1930.8 (834.3)	4097.3 (554.4)	6258.7 (731.8)	11 077.4 (3664.4)
Dietary intake, mean (SD)					
Total, kcal/d	2424.9 (1338.6)	2257.3 (1293.5)	2380.4 (1301.5)	2462.8 (1373.0)	2599.1 (1364.7)
Saturated fat, % kcal/d	11.6 (2.5)	11.5 (2.2)	11.6 (2.3)	11.7 (2.2)	11.5 (3.0)
Vegetables, servings/d	2.7 (2.1)	2.5 (2.0)	2.7 (2.0)	2.7 (2.3)	2.7 (2.1)
Fruit, servings/d	1.0 (0.9)	1.1 (0.9)	1.0 (0.8)	1.0 (0.9)	1.1 (0.9)
Alternative Healthy Eating Index score	44.6 (9.0)	45.4 (8.4)	44.6 (8.8)	44.2 (9.0)	44.1 (9.9)

Abbreviations: BMI, body mass index (calculated as body weight in kilograms divided by height in meters squared); CVD, cardiovascular disease; HDL, high-density lipoprotein; LDL, low-density lipoprotein.

SI conversion factors: To convert cholesterol to mmol/L, multiply values by 0.0259. To convert waist circumference to cm, multiply values by 2.54.

^a^ Unless indicated otherwise, values are presented as No. (%) of participants.

**Figure 1.**
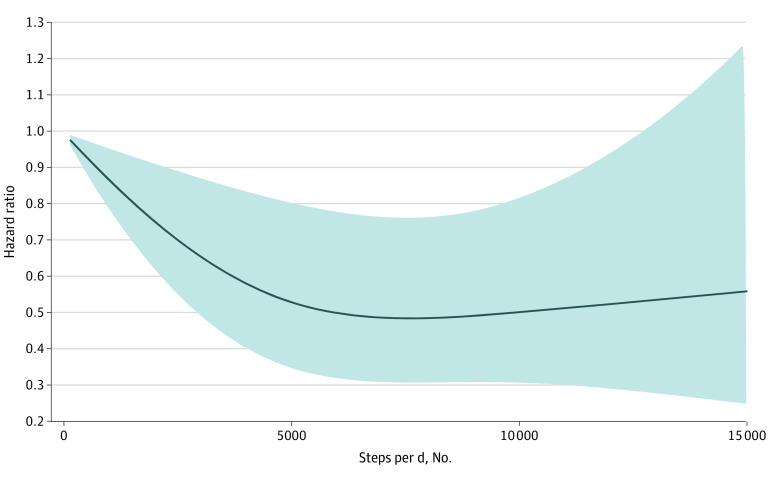
Restricted Cubic Spline Models of Hazard Ratios of Steps per Day and All-Cause Mortality Model adjusted for age, sex, study site, education, smoking status, alcohol use and diet quality (both as assessed with the Alternative Healthy Eating Index), body mass index, systolic blood pressure, prevalent diabetes, prevalent cardiovascular disease, biomarker levels (fibrinogen, low-density lipoprotein cholesterol, and triglycerides), medication use (hypertensive or lipid-lowering agents), and self-reported health status.

**Figure 2.**
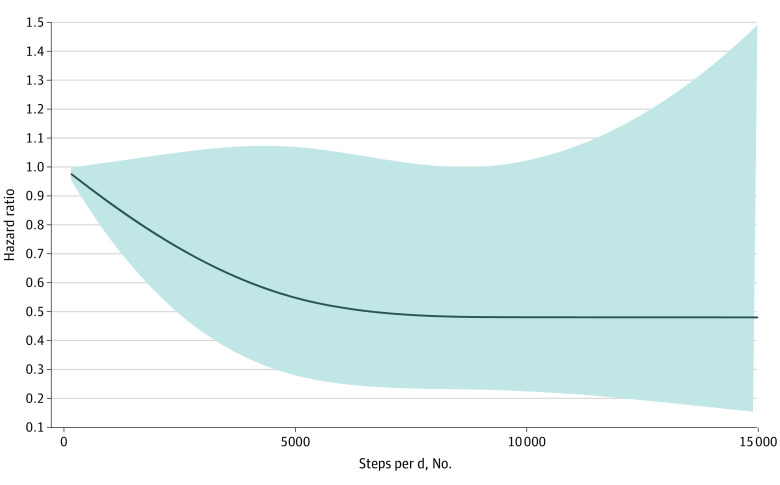
Restricted Cubic Spline Models of Hazard Ratios of Steps per Day and Cardiovascular Disease Mortality Model adjusted for age, sex, study site, education, smoking status, alcohol use and diet quality (both as assessed with the Alternative Healthy Eating Index), body mass index, systolic blood pressure, prevalent diabetes, prevalent cardiovascular disease, biomarker levels (fibrinogen, low-density lipoprotein cholesterol, and triglycerides), medication use (hypertensive or lipid-lowering agents), and self-reported health status.

During a mean follow-up of 17.0 years (range, 0-19.9 years), there were 449 deaths (123 were attributable to CVD). Participants in the upper 3 quartiles of steps per day had a 28.0% to 35.0% lower risk of death than participants in the lowest quartile of steps per day. The hazard ratios for mortality among those in the upper 3 quartiles of steps per day were 0.72 (95% CI, 0.54-0.95) for the first quartile, 0.66 (95% CI, 0.47-0.93) for the second quartile, and 0.65 (95% CI, 0.44-0.95) for the third quartile compared with participants in the lowest quartile after adjustment for age, sex, study site, education, smoking status, alcohol use, diet quality, BMI, systolic blood pressure, prevalent diabetes, prevalent CVD, biomarker levels (fibrinogen, LDL cholesterol, and triglycerides), medication use (hypertensive or lipid-lowering agents), and self-reported health status (Table 2). The use of cubic spline models to examine the association of steps per day and risk of death did not materially alter the hazard ratios (Figure 1). Associations appeared similar when analyses were repeated using CVD mortality as the outcome (Figure 2 and Table 3).

**Table 2.** Hazard Ratios for Total Mortality According to Ambulatory Activity Quartile^a^

	Ambulatory activity quartile, steps/d (N = 2204)			
	<3126 (n = 188)	3126-5085 (n = 117)	5086-7572 (n = 76)	≥7573 (n = 68)
Person-years at risk	8581.3	9335.6	9729.0	9778.9
Model 1^b^	1 [Reference]	0.71 (0.58-0.88)	0.52 (0.40-0.70)	0.52 (0.39-0.69)
Model 2^c^	1 [Reference]	0.74 (0.59-0.91)	0.54 (0.41-0.71)	0.54 (0.41-0.71)
Model 3^d^	1 [Reference]	0.72 (0.54-0.95)	0.66 (0.47-0.93)	0.65 (0.44-0.95)

^a^ Unless indicated otherwise, values are presented as hazard ratios (95% CIs).

^b^ Model 1 adjusted for age, sex, and study site.

^c^ Model 2 additionally adjusted for education, smoking status, and alcohol use and diet quality (both as assessed with the Alternative Healthy Eating Index).

^d^ Model 3 additionally adjusted for body mass index, systolic blood pressure, prevalent diabetes, prevalent cardiovascular disease, biomarker levels (fibrinogen, low-density lipoprotein cholesterol, and triglycerides), medication use (hypertensive or lipid-lowering agents), and self-reported health status.

**Table 3.** Hazard Ratios for Cardiovascular Mortality According to Ambulatory Activity Quartile^a^

	Ambulatory activity quartile, steps/d (N = 2204)			
	<3126 (n = 49)	3126-5085 (n = 37)	5086-7572 (n = 19)	≥7573 (n = 18)
Person-years at risk	8581.3	9335.6	9729.0	9778.9
Model 1^b^	1 [Reference]	0.89 (0.56-1.41)	0.53 (0.34-0.82)	0.57 (0.32-0.99)
Model 2^c^	1 [Reference]	0.95 (0.59-1.51)	0.56 (0.36-0.89)	0.57 (0.32-1.01)
Model 3^d^	1 [Reference]	1.12 (0.70-1.78)	0.68 (0.42-1.46)	0.78 (0.42-1.46)

^a^ Cardiovascular mortality includes deaths from myocardial infarction, coronary heart disease, heart failure, or stroke. Unless indicated otherwise, values are presented as hazard ratios (95% CIs).

^b^ Model 1 adjusted for age, sex, and study site.

^c^ Model 2 additionally adjusted for education, smoking status, and alcohol use and diet quality (both as assessed with the Alternative Healthy Eating Index).

^d^ Model 3 (primary model) additionally adjusted for body mass index, systolic blood pressure, prevalent diabetes, prevalent cardiovascular disease, biomarker levels (fibrinogen, low-density lipoprotein cholesterol, and triglycerides), mediation use (hypertensive or lipid-lowering agents), and self-reported health status.

There was no statistically significant interaction of ambulatory activity with age (*P* = .21), sex (*P* = .32), study site (*P* = .32), BMI (*P* = .36), or diabetes status (*P* = .91) (hazard ratio, 1.00 [95% CI, 0.99-1.00] for all) on risk of death. Sensitivity analyses that (1) omitted the days with the minimum and maximum accumulated steps in estimating the mean steps per day or (2) excluded the 24 participants who died during the first 2 years of follow-up did not materially alter the reported hazard ratios (eTables 1 and 2 in Supplement 1). Exploratory analyses that omitted participants with self-reported fair or poor health at baseline or with prevalent CVD produced generally similar results (eTable 3 in Supplement 1).

## Discussion

The findings of this large cohort study of American Indian individuals suggest that ambulatory activity (eg, walking) is associated with a lower risk of death. Participants in the upper 3 quartiles of ambulatory activity had a 28.0% to 35.0% lower risk of death compared with participants in the lowest quartile of activity.

Our results support findings from 2 prospective US studies that suggest an inverse association of ambulatory activity with risk of death among young and middle-aged adults. In the Coronary Artery Risk Development in Young Adults (CARDIA) study, participants who accumulated at least 7000 steps/d had a 50% to 70% lower risk of death compared with those who accumulated fewer steps per day; accumulation of more than 10 000 steps/d was not associated with further reductions in risk of death.^19^ Similarly, in a study using data from the National Health and Nutrition Examination Survey (NHANES; activity assessed in 2003-2006; mortality follow-up through December 2015), participants who accumulated 8000 steps/d had a 51% lower risk of death compared with participants who accumulated 4000 steps/d.^33^ In the NHANES study, hazard ratios plateaued at about 10 000 steps/d, and taking more than 10 000 steps/d was not associated with further reduction in risk.^33^ In the SHFS, hazard ratios were similar across the upper 2 quartiles of steps per day. Differences in hazard ratios reported across cohorts may be, in part, explained by differences in the distribution (and subsequent analytic categorization) of ambulatory activity levels. Although each study focused on young and/or middle-aged populations (with mean ages of 41.0, 45.2, and 56.8 years in the SHFS, CARDIA, and NHANES studies), reported levels of ambulatory activity in the CARDIA and NHANES studies were strikingly higher than those in the SHFS. A total of 16.6% of participants in the SHFS accumulated more than 9100 steps/d (median, 5085 steps/d), while more than 50% of participants in the CARDIA and NHANES studies accumulated at least 9100 steps/d (median, 9146 vs 9124 steps/d).^19,33^

Our results are also consistent with findings from studies that have focused on older adults.^13,14,15,16,17,18^ These studies primarily comprised older populations in Europe, Australia, and Asia, and to our knowledge, only 1 published study has focused on older adults in the US. Among women aged 62 to 101 years who participated in the Women’s Health Study (WHS), accumulation of 4400 steps/d was associated with lower risk of death compared with lower levels of activity.^18^ Risk of death declined with an increased number of daily steps up to about 7500 steps/d. Above 7500 steps/d, hazard ratios for death remained constant.^18^ Although the WHS participants were older than the SHFS participants (mean age, 72.0 vs 41.0 years), the mean number of steps per day was similar in the 2 cohorts (5841 vs 5085 steps/d).

Published studies report that the influence of steps per day on risk of death is nonlinear, and only marginal benefit is achieved beyond a specific threshold—typically 6000 to 10 000 steps/d.^11,34^ A recent meta-analysis of 15 studies reported little benefit on mortality risk beyond 6000 to 8000 steps/d among older adults and 8000 to 10 000 steps/d among younger adults (aged <60 years).^11^ The authors hypothesized that activity levels needed to improve health differ by age. However, the low threshold observed in the SHFS—with a young and largely inactive population—suggests that other biological factors should be considered, including underlying CVD risk, genetics, and resting metabolic rate. Better understanding of the role of different activity thresholds in health is needed to inform clinical CVD risk reduction programs and personalize medical advice.

Elucidation of the association of ambulatory activity with risk of death is challenging in observational studies due to the potential impact of underlying morbidity on risk estimates. It is possible that lower levels of activity cause morbidity (eg, CVD or diabetes), which increases risk of death. In this case, models that adjust for underlying morbidity may underestimate the association of ambulatory activity with risk of death, and adjusting for morbidity may be considered an overadjustment. Alternatively, it is possible that morbidity may confound the association of activity with risk of death because individuals with morbid conditions generally have higher mortality risk than healthy individuals and may also have different activity patterns. We cannot differentiate between mediation and confounding in this observational study. For the primary analyses, we decided a priori to include all SHFS participants irrespective of underlying morbidity and health status to increase comparability with other studies among community-dwelling adults that used similar inclusion and/or exclusion criteria.^11,13,14,15,16,17,18,19,33,34,35,36,37^ It is reassuring that the observed associations persisted across models 2 and 3, suggesting that they cannot be completely explained by underlying morbidity.

### Strengths and Limitations

Our study has several strengths. To our knowledge, it is the only study of ambulatory activity and risk of death among American Indian individuals—a population with high risk of CVD and lower life expectancy than the general US population.^38^ The use of pedometers to measure activity may more accurately capture walking (ie, the most common activity in the SHFS^20^) and other unstructured ambulatory activities common among American Indian individuals that are difficult to self-report on questionnaires. Other strengths of this study include the availability of high-quality data on a variety of risk factors, including diet and biomarkers.

This study also has limitations. Although activity was assessed using an objective measurement, it is possible that study participants altered their activity patterns during the days that the pedometer was worn or did not accurately record steps in their diaries. However, altered activity or inaccurate step recording is not likely to differ by future risk of death; this nondifferential misclassification would bias our reported risk estimates toward the null. Additionally, pedometers are designed to only capture ambulatory movement, such as walking, and activities such as swimming, biking, or lifting are not captured. Because pedometers only detect the number of steps taken and do not discriminate between activities, they are unable to measure activity intensity. However, recent work has shown that volume of steps is a better estimator of risk of death than step intensity.^18,19,33^

## Conclusions

Step counters constitute an inexpensive tool that offers the opportunity to encourage activity and improve long-term health outcomes. The findings of this cohort study add to the growing body of evidence identifying physical inactivity as an important factor associated with mortality and suggests the need for physical activity outreach programs that target inactive individuals.

## Notes

Supplement 1. **eTable 1.** Hazard Ratios for Total Mortality According to Ambulatory Activity Quartile (Analyses Exclude Days With Minimum and Maximum Accumulated Steps) (n=2204) **eTable 2.** Hazard Ratios for Total Mortality According to Ambulatory Activity Quartile (Excluding Participants Who Died During the First 2 Years of Follow-up) (n=2180) **eTable 3.** Hazard Ratios for Total Mortality According to Ambulatory Activity Quartile (Restricted to Participants With Self-reported Health Status of Excellent/Very Good/Good and Without Prevalent Cardiovascular Disease at Baseline) (n=1655)

Supplement 2.
Data Sharing Statement
