# Supplementary material for: Ambulatory Activity and Risk of Premature Mortality Among Young and Middle-aged American Indian Individuals
Source: JAMA Netw Open. 2023 May 4;6(5):e2311476. doi: 10.1001/jamanetworkopen.2023.11476 (PMC10160874; doi:10.1001/jamanetworkopen.2023.11476)
Supplement: Supplement 1. — eTable 1. Hazard Ratios for Total Mortality According to Ambulatory Activity Quartile (Analyses Exclude Days With Minimum and Maximum Accumulated Steps) (n=2204) eTable 2. Hazard Ratios for Total Mortality According to Ambulatory Activity Quartile (Excluding Participants Who Died During the First 2 Years of Follow-up) (n=2180) eTable 3. Hazard Ratios for Total Mortality According to Ambulatory Activity Quartile (Restricted to Participants With Self-reported Health Status of Excellent/Very Good/Good and Without Prevalent Cardiovascular Disease at Baseline) (n=1655) [file jamanetwopen-e2311476-s001.pdf]

Supplementary Online Content

Fretts AM, Siscovick DS, Malloy K, et al. Ambulatory activity and risk of premature mortality among young and middle-aged American Indian individuals. *JAMA Netw Open*. 2023;6(5):e2311476. doi:10.1001/jamanetworkopen.2023.11476

eTable 1. Hazard Ratios for Total Mortality According to Ambulatory Activity Quartile (Analyses Exclude Days With Minimum and Maximum Accumulated Steps) (n=2204)

eTable 2. Hazard Ratios for Total Mortality According to Ambulatory Activity Quartile (Excluding Participants Who Died During the First 2 Years of Follow-up) (n=2180)

eTable 3. Hazard Ratios for Total Mortality According to Ambulatory Activity Quartile (Restricted to Participants With Self-reported Health Status of Excellent/Very Good/Good and Without Prevalent Cardiovascular Disease at Baseline) (n=1655)

This supplementary material has been provided by the authors to give readers additional information about their work.

© 2023 Fretts AM et al. *JAMA Network Open*.

**eTable 1.** Hazard Ratios for Total Mortality According to Ambulatory Activity Quartile (Analyses Exclude Days With Minimum and Maximum Accumulated Steps) (n=2204)				
**Steps/day**	**<3097**	**3097-5003**	**5004-7617**	**7618+**
**Cases**	185	113	86	65
**Person-Years at Risk**	8585.0	9391.5	9615.4	9832.9
**Model 1[a]**	1.0 (ref)	0.70 (0.57, 0.87)	0.64 (0.48, 0.84)	0.51 (0.39, 0.67)
**Model 2[b]**	1.0 (ref)	0.73 (0.59, 0.91)	0.65 (0.50, 0.86)	0.53 (0.40, 0.69)
**Model 3[c]**	1.0 (ref)	0.79 (0.63, 0.99)	0.74 (0.57, 0.97)	0.63 (0.48, 0.84)

[a] Model 1 adjusted for age, sex, study site; [b] model 2 additionally adjusted for education, smoking, alcohol, and diet quality (as assessed with the Alternative Healthy Eating Index); [c] model 3 additionally adjusted for BMI, systolic blood pressure, prevalent diabetes, fibrinogen, LDL cholesterol, triglycerides, hypertension medication use, lipid-lowering drug use

**eTable 2.** Hazard Ratios for Total Mortality According to Ambulatory Activity Quartile (Excluding Participants Who Died During the First 2 Years of Follow-up) (n=2180)				
**Steps/day**	**<3126**	**3126-5085**	**5086-7572**	**7573+**
**Cases**	178	111	71	65
**Person-Years at Risk**	8570.8	9328.5	9724.5	9775.6
**Model 1[a]**	1.0 (ref)	0.71 (0.57, 0.88)	0.52 (0.38, 0.69)	0.53 (0.40, 0.70)
**Model 2[b]**	1.0 (ref)	0.73 (0.59, 0.91)	0.53 (0.40, 0.71)	0.54 (0.41, 0.71)
**Model 3[c]**	1.0 (ref)	0.71 (0.54, 0.94)	0.66 (0.45, 0.95)	0.66 (0.45, 0.98)

[a] Model 1 adjusted for age, sex, study site; [b] model 2 additionally adjusted for education, smoking, alcohol, and diet quality (as assessed with the Alternative Healthy Eating Index); [c] model 3 additionally adjusted for BMI, systolic blood pressure, prevalent diabetes, fibrinogen, LDL cholesterol, triglycerides, hypertension medication use, lipid-lowering drug use

**eTable 3.** Hazard Ratios for Total Mortality According to Ambulatory Activity Quartile (Restricted to Participants With Self-reported Health Status of Excellent/Very Good/Good and Without Prevalent Cardiovascular Disease at Baseline) (n=1655)				
**Steps/day**	**<3126**	**3126-5085**	**5086-7572**	**7573+**
**Cases**	84	85	53	51
**Person-Years at Risk**	5366.5	7016.3	8144.6	8313.9
**Model 1[a]**	1.0 (ref)	0.92 (0.67, 1.26)	0.55 (0.38, 0.80)	0.57 (0.40, 0.81)
**Model 2[b]**	1.0 (ref)	0.96 (0.70, 1.31)	0.57 (0.40, 0.82)	0.57 (0.40, 0.82)
**Model 3[c]**	1.0 (ref)	1.00 (0.72, 1.40)	0.60 (0.41, 0.88)	0.62 (0.43, 0.91)

[a] Model 1 adjusted for age, sex, study site; [b] model 2 additionally adjusted for education, smoking, alcohol, and diet quality (as assessed with the Alternative Healthy Eating Index); [c] model 3 additionally adjusted for BMI, systolic blood pressure, prevalent diabetes, fibrinogen, LDL cholesterol, triglycerides, hypertension medication use, lipid-lowering drug use

© 2023 Fretts AM et al. *JAMA Network Open*.
