# Supplementary material for: Ambulatory Activity and Risk of Premature Mortality Among Young and Middle-aged American Indian Individuals
Source: JAMA Netw Open. 2023 May 4;6(5):e2311476. doi: 10.1001/jamanetworkopen.2023.11476 (PMC10160874; doi:10.1001/jamanetworkopen.2023.11476)
Supplement: Supplement 2. — Data Sharing Statement [file jamanetwopen-e2311476-s002.pdf]

Data Sharing Statement

Fretts. Ambulatory Activity and Risk of Premature Mortality Among Young and Middle-Aged American Indian Individuals. *JAMA Netw Open*. Published May 04, 2023. doi:10.1001/jamanetworkopen.2023.11476

Data
Data available: No

Additional Information
Explanation for why data not available: Data described in the manuscript and/or analytic code will not be made available publicly due to data sharing agreements with participating American Indian tribes (the authors are not authorized to share data). Details on how to request access to the Strong Heart Study data are located on the study website: https://strongheartstudy.org/
